# Association between physical education classes and physical activity among 187,386 adolescents aged 13–17 years from 50 low- and middle-income countries

**DOI:** 10.1016/j.jped.2020.11.009

**Published:** 2021-02-05

**Authors:** Xuzhi Zhan, Cain C.T. Clark, Ran Bao, Micheal Duncan, Jin-Tao Hong, Si-Tong Chen

**Affiliations:** a School of Physical Education and Humanity, Nanjing Sport Institute, Jiangsu, China; b Centre for Intelligent Healthcare, Coventry University, Coventry, United Kingdom; c School of Physical Education and Sports Training, Shanghai University of Sport, Shanghai, China; d Centre for Sport, Exercise and Life Sciences, Coventry University, Coventry, United Kingdom; e Shanghai Research Institute of Sports Science, Shanghai, China; f Institute for Health and Sport, Victoria University, Melbourne, Australia

**Keywords:** Physical activity epidemiology, Physical education, GSHS, Adolescents, Low- and middle-income countries

## Abstract

**Objective:**

This study aimed to examine the association between physical education classes and PA among adolescents from 50 low- and middle-income countries (LMICs).

**Methods:**

A self-reported questionnaire from the Global School-based Student Survey (GSHS) was used to collect information on participation frequency of physical education classes and being physically active over the last week, as well as other control variables (e.g., sex, age, country, sedentary behavior). Multivariable logistic regression and a pooled meta-analysis were performed to explore the association and compared country-wise differences.

**Results:**

Included adolescents aged from 13 to 17 years (n = 187,386, %boys = 51.7; mean age = 14.6 years), the prevalence of sufficient PA (meeting the PA guidelines) was 14.9%. The prevalence of 5 days or more to engage in physical education classes was 16.5%. Compared with adolescents who had 0 days for physical education classes, higher participation frequency was more likely related to sufficient PA (OR: 1 day = 1.34, 2 days = 1.66, 3 days = 1.67, 4 days = 1.79, 5 days or more = 2.46), these findings were also observed in both sexes. A moderate inconsistency on the association across the included countries was found (*I^2^* = 53%, p < 0.01), although the pooled OR was 1.50 (95% CI: 1.36–1.65).

**Conclusions:**

Participating in more physical education classes may be an effective approach to increase physical activity levels among adolescents in LMICs. However, promoting physical activity levels among adolescents in LMICs through physical education classes should consider more country-specific factors.

## Introduction

Sufficient physical activity (PA) is regarded as an important factor in promoting physical and mental health outcomes, like improving aerobic fitness, promoting cognitive developments, reducing adiposity rate and preventing depressive symptoms.^1^, ^2^, ^3^ Many health organizations recommend attaining a minimum of 60 min of moderate to vigorous PA per day, for children and adolescents aged 5–17 years.^4^, ^5^ However, many large-scale surveys report that children and adolescents do not accrue sufficient PA. An international study, including 1.6 million adolescents, reported that less than 20% of adolescents were physically active.^6^ Among Chinese and U.S. children and adolescents, the prevalence of having sufficient PA (at least 60 min of moderato to vigorous PA per day) was less than 20%.^7^, ^8^ Indeed, low levels of PA are commonly reported by various countries, especially in low and middle-income countries (LMICs).^9^, ^10^, ^11^ In the face of this health issue, encouraging children and adolescents physical activity participation is an urgent action.^12^

In the school setting, physical education classes have been proposed as a possible avenue to increase PA, because physical education classes play a role in shaping children's and adolescents’ active lifestyles.^10^ Dishman et al.^13^ posited that the practice of PA leads to changes at the level of the brain that stimulates vigor and more movement. Accordingly, young people engaging in physical education classes appear to be more physically active than their non-participatory counterparts.^14^ Although the mechanism of why physical education classes are associated with increased PA has not yet been fully understood, the use of physical education classes to promote PA in adolescents has been advocated.^15^ Some prior empirical research has examined the association between physical education classes and PA among children and adolescents, and in a study including adolescents from 12 countries it was reported that male children, aged 9–11 years, who had more than 3 days of physical education classes per week, were more likely meet the PA guidelines (OR = 1.62, 95%CI: 1.17–2.24), as compared with those with 0 days of physical education classes.^16^ However, counterintuitively, this significant association did not exist in girls.^16^ Furthermore, this study also examined the association in the adolescents from LMICs and indicated that children who undertook more physical education classes were more likely to meet the PA guidelines, regardless of gender.^16^ A study by Chen et al.^17^ also supported the positive role of physical education classes in promoting PA in children, suggesting that a one-minute increase in moderate to vigorous physical activity (MVPA) in physical education classes was associated with an increase of 2.04 min in daily MVPA. In addition to the large-scale evidence reported above, there have been many empirical studies confirming the positive relationship between physical education classes and PA levels among children and adolescents.^18^, ^19^

However, despite the potential utility of school physical education to enhance PA, there are limitations in prior research which should be mentioned and also lays the foundation for the current study. One major gap is that previous studies systematically lack sufficient evidence on the association between physical education classes and PA in adolescents from LMICs.^10^, ^16^ Another study gap is the evidence from LMICs comes from comparatively small-scale studies,^16^ with some studies that do not support the role of physical education classes in promoting PA among adolescents.^16^ Collectively, it is evident that adequate adolescents from LMICs are urgently needed to discern the associations between physical education classes and PA among adolescents. Drawing inferences on the impact of physical education on PA, based on studies from high-income countries (HICs) may lead to erroneous conclusions being drawn as the environmental constraints differ considerably between HICs and LMICs. Moreover, previously published research has concentrated on children or adolescents with a narrower age range (e.g., 9–11 years),^16^ impeding our understanding of the association between physical education classes and PA. Lastly, there is still no global study of adolescents from LMICs. Indeed, this type of study can help inform PA promotion for adolescents with socioeconomic disparities.^20^

Therefore, this study sought to examine the association between physical education classes and PA, based on adolescents from 50 LMICs, using the Global School-based Health Survey (GSHS) data.

## Methods

### Study survey

In this cross-sectional study, we utilized publicly available data of GSHS survey collected between 2009 and 2017 (available time period). The GSHS is an international epidemiologic surveillance aiming at assessing and quantifying the risk and protective factors of major non-communicable diseases among school-attending adolescents. Using a standardized two-stage probability sampling design, representative of all students within each participating country were selected. Specifically, schools were chosen with probability proportional to size sampling in the initial stage, and the random selection of classrooms and students within each selected school were performed in the second stage. All eligible students completed a standardized and questionnaire in local language with their responses on computer scannable sheets. Before the conduction of the GSHS surveys, approvals were obtained from both a national government administration (education or health relevant ministry) and an institutional review board or ethics committee in each country. Informed consent were also received, as appropriate, from the students, parents, and/or school officials. According to the World Bank classification of countries by income at the survey time, fifty eligible LMICs were included. Data were weighted for non-response and probability selection, and we selected datasets containing the variables pertaining to this present study. We retained the latest dataset if there were more than one dataset from the same country from 2009 to 2017. The sample is comprised of 187386 adolescents in total, with ages ranging from 13 to 17 years.^21^

## Measures

### Physical education classes (exposure)

One question was used to assess physical education classes frequency among adolescents. The question was: during this school year, on how many days did you go to physical education class each week? The responses ranged from 1 = 0 days, to 6 = 5 or more days.

### Physical activity (outcome)

Physical activity was assessed with the question from the PACE + Adolescent Physical Activity Measure, which asks adolescents to report the number of days with physical activity of at least 60 min during the past 7 days (from none = 0 days to 8 = 7 days). This measure has been tested for acceptable validity and reliability.^22^ Based on global health behavior guidelines, adolescents reporting 7 days were regarded as meeting the physical activity guidelines.^4^, ^5^

### Covariates

Variables of sex and age were selected as control variables in this study. These variables were measured by the self-reported questionnaire, which asked for confirmation of sex (boy or girl), age (11 and younger, 12, 13, 14, 15, 16, 17 and 18 years and older). Sedentary behavior was assessed with the question, “How much time do you spend during a typical or usual day sitting and watching television, playing computer games, talking with friends, or doing other sitting activities?”, with answer options: <1, 1–2, 3–4, 5–6, 7–8, and ≥8 h/day. This excluded time at school and when doing homework. To measure study participants’ socioeconomic status, a proxy report measure, which was food insecurity, was used in this study. Food insecurity was assessed by one question “During the past 30 days, how often did you go hungry because there was not enough food in your home?” Answer options were in five categories: “never”, “rarely”, “sometimes”, “most of the time” and “always”.

## Statistical analyses

All were analyses were restricted to adolescents aged 13–17 years, concordant with our research aim. Descriptive statistics of percentages were used to report the levels of study exposure (physical education classes frequency) and outcomes (the prevalence of meeting the PA guidelines). Inferential statistical was used to compare differences in the prevalence of physical education classes frequency and meeting the PA guidelines across the different groups. To estimate population-based descriptive results of study exposure and outcomes, sampling weight and complex study design was used, which generated the weighed prevalence of days for physical education classes, the prevalence of meeting the PA guidelines, and the prevalence of sedentary behavior under 3 h per day. Multivariable logistic regression was used to assess the association of physical education classes with PA. To assess the country-based heterogeneity of the association between physical education with physical activity, the Higgin’s *I*^2^ statistic was calculated. The value of <40% of Higgin’s *I*^2^ represents negligible heterogeneity while 40–60% as moderate heterogeneity.^23^ This estimation was achieved using a meta-analysis with a random effect model. All variables included in our study were treated as categorical variables (except for age) when performing logistic regression model. Taylor linearization methods were used in all analyses, taking complex survey sampling into account. Results of the logistic analysis models were displayed using odds ratios (ORs) at 95% confidence intervals (CIs). We considered p < 0.05 as significantly statistical level at two-sided.

## Results

Table 1 shows sample characteristics by each country, of all the included 187 386 participants in which boys accounted for 51.7%, and the mean age was 14.6 ± 1.2 years. Table 1 also shows the prevalence of physical education classes frequency and the prevalence of meeting the PA guidelines. Specifically, in the total sample, the prevalence of physical education classes frequency varied greatly (0 days = 21.1%, 1 day = 36.1%, 2 days = 18.7%, 3 days = 4.7%, 4 days = 2.9%, 5 or more days = 16.5%). The prevalence of meeting the PA guidelines was 14.9%, and the highest prevalence in each country was 42.3% (Bangladesh; being higher than others, p < 0.001).

**Table 1:** Sample characteristics of the survey study.

Country	Sample size	Year	0 days	1 day	2 days	3 days	4 days	5 days or more	Physical activity^a^
Total	187,386	/	21.1%	36.1%	18.7%	4.7%	2.9%	16.5%	14.9%
Afghanistan	1809	2014	31.1%	19.4%	11.5%	10.7%	9.6%	17.7%	9.2%
Algeria	3687	2011	15.7%	55.1%	6.0%	4.0%	3.0%	16.3%	15.8%
Antigua & Barbuda	1066	2009	28.3%	40.3%	5.7%	2.6%	2.2%	20.9%	22.8%
Argentina	24,932	2012	7.9%	15.9%	50.9%	2.8%	2.4%	20.1%	16.6%
Bangladesh	2603	2014	10.5%	10.0%	30.1%	15.6%	6.2%	27.6%	42.3%
Belize	1482	2011	28.2%	42.6%	9.1%	4.1%	2.7%	13.3%	20.7%
Benin	1511	2016	13.1%	66.4%	8.6%	2.4%	1.2%	8.3%	29.3%
Bolivia	3161	2012	12.1%	51.2%	5.2%	2.1%	1.9%	27.6%	14.2%
Cambodia	2761	2013	35.3%	36.9%	17.0%	3.7%	1.4%	5.7%	6.8%
Costa Rica	2544	2009	20.3%	43.6%	2.3%	1.8%	2.1%	29.9%	18.3%
Dominican Republic	1203	2016	22.1%	24.3%	20.8%	4.3%	3.8%	24.6%	13.3%
Egypt	1959	2011	35.0%	25.9%	22.3%	3.0%	0.9%	13.0%	12.6%
El Salvador	1736	2013	12.9%	34.7%	14.0%	1.6%	1.9%	34.9%	13.1%
Fiji	2789	2016	19.0%	48.9%	10.7%	5.8%	4.3%	11.3%	20.7%
Ghana	2115	2012	27.4%	29.1%	14.5%	7.8%	4.8%	16.3%	12.1%
Guatemala	3565	2015	10.3%	41.2%	12.4%	2.7%	4.6%	28.7%	10.7%
Guyana	2207	2010	49.9%	22.4%	8.9%	4.8%	2.5%	11.3%	15.6%
Honduras	1399	2012	9.5%	38.1%	22.9%	2.0%	2.0%	25.5%	15.9%
Indonesia	8553	2015	11.3%	66.2%	10.2%	2.7%	0.8%	8.9%	12.9%
Iraq	1720	2012	35.2%	21.3%	13.7%	4.0%	2.6%	23.3%	15.2%
Jamaica	1475	2017	40.0%	35.7%	5.6%	3.4%	1.6%	13.8%	22.9%
Kiribati	1453	2011	21.5%	30.7%	13.7%	6.5%	3.1%	24.6%	17.9%
Lao People’s Democratic Republic	3552	2015	32.8%	54.3%	2.4%	0.9%	0.9%	8.7%	16.6%
Lebanon	4148	2017	41.1%	27.6%	9.5%	2.9%	1.9%	16.9%	12.8%
Liberia	998	2017	30.8%	27.4%	12.9%	4.0%	4.1%	20.8%	10.4%
Malaysia	24,684	2012	11.1%	42.3%	20.6%	3.3%	2.2%	20.5%	14.2%
Mauritania	1721	2010	41.8%	19.4%	8.9%	3.8%	4.4%	21.7%	12.5%
Mauritius	2770	2017	16.7%	49.2%	6.0%	3.9%	3.3%	20.8%	18.9%
Mongolia	4395	2013	4.6%	25.6%	63.5%	2.5%	0.4%	3.5%	24.8%
Morocco	4507	2016	18.9%	12.9%	33.3%	4.4%	2.9%	27.7%	10.6%
Mozambique	1167	2015	8.4%	33.6%	36.0%	7.0%	5.5%	9.5%	13.1%
Myanmar	2387	2016	31.6%	33.1%	17.4%	5.5%	2.4%	9.9%	10.2%
Namibia	3092	2013	24.0%	39.2%	7.5%	3.1%	3.1%	23.1%	13.7%
Nepal	5360	2015	32.7%	13.4%	9.6%	7.6%	5.7%	30.9%	15.5%
Pakistan	9796	2009	59.5%	12.2%	14.3%	4.2%	1.9%	7.9%	11.3%
Paraguay	2611	2017	14.7%	59.3%	6.2%	2.7%	1.6%	15.4%	17.1%
Peru	2783	2010	7.7%	86.0%	4.0%	0.6%	0.1%	1.5%	15.2%
Philippines	7574	2015	13.5%	29.8%	9.9%	5.6%	7.4%	33.7%	7.6%
Samoa	1817	2011	37.5%	23.1%	11.5%	8.1%	5.1%	14.6%	12.1%
Solomon Islands	1154	2011	27.5%	24.9%	8.1%	5.0%	6.0%	28.5%	16.9%
Sri Lanka	3075	2016	30.6%	13.4%	14.6%	10.4%	6.6%	24.4%	15.1%
Sudan	1947	2012	53.2%	24.4%	7.1%	4.2%	1.7%	9.3%	7.9%
Suriname	1697	2016	18.4%	42.3%	5.6%	3.7%	1.7%	28.3%	19.3%
Syrian Arab Republic	2486	2010	20.6%	48.6%	5.7%	2.2%	1.8%	21.1%	11.0%
Tanzania	2743	2014	35.3%	16.8%	10.6%	6.8%	5.7%	24.8%	19.8%
Thailand	4809	2015	14.1%	66.2%	9.5%	3.1%	1.1%	6.1%	11.6%
Timor-Leste	2591	2015	15.9%	45.5%	12.4%	3.7%	2.8%	19.7%	9.7%
Tonga	2279	2017	53.3%	15.4%	8.7%	4.7%	3.4%	14.4%	18.0%
Tuvalu	636	2013	44.0%	10.6%	8.5%	4.1%	4.7%	28.0%	11.0%
Vietnam	2976	2013	3.5%	43.5%	48.6%	1.1%	0.5%	2.8%	13.1%
Yemen	1901	2014	53.7%	13.7%	6.5%	2.9%	3.0%	20.2%	11.5%

a Denotes meeting the physical activity guidelines the results in this Table were weighted by complex samples.

**Figure 1:**
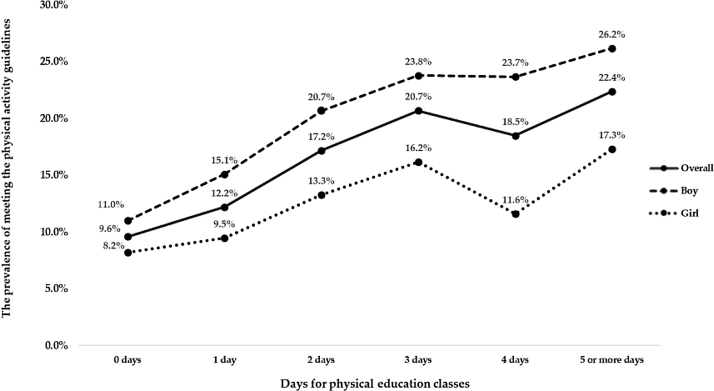
The prevalence of meeting the physical activity guidelines by days of physical education classes frequency.

The prevalence of meeting the PA guidelines by days for physical education classes frequency is shown in Fig. 1. Generally, the prevalence of meeting the PA guidelines increased with more days for physical education classes frequency, regardless of sex. However, there was a turning point for the prevalence of meeting the PA guidelines; where adolescents with 4 days of physical education classes frequency had a lower prevalence of meeting the PA guidelines, as compared with peers who had 3 days of physical education classes frequency (p < 0.001), irrespective of sex.

Results of the associations of physical education classes frequency with PA among adolescents are shown in Table 2. In the multivariable regression model, compared with adolescents without physical education classes frequency, those who had more days for physical education classes frequency were more likely to meet the PA guidelines, regardless of sex (all ORs > 1). With higher physical education classes frequency, the odds ratio of meeting the PA guidelines increased consistently (OR for 1 day = 1.34, 2 days = 1.66, 3 days = 1.67, 4 days = 1.79, 5 or more days = 2.46). This significant trend was also observed in both boys and girls (Table 2).

**Table 2:** Association between physical education classes and meeting the physical activity guidelines estimated by multivariable logistic regression (overall and by sex).

	Overall^a^			Boy^b^			Girl^b^		
Physical education classes	OR	95% CI		OR	95% CI		OR	95% CI	
0 days	Ref			Ref			Ref		
1 day	1.34	1.16	1.55	1.44	1.18	1.75	1.20	1.01	1.42
2 days	1.66	1.35	2.05	1.74	1.29	2.35	1.53	1.23	1.90
3 days	1.67	1.38	2.02	1.78	1.38	2.30	1.52	1.18	1.94
4 days	1.79	1.38	2.31	2.07	1.48	2.89	1.38	1.02	1.93
5 or more days	2.46	2.14	2.83	2.65	2.17	3.22	2.20	1.84	2.64

OR, odds ratio; CI, confidence interval; Ref, reference group.

a Denote controlling for sex, age, food insecurity, sedentary behavior and country.

b Denotes controlling for age, food insecurity, sedentary behavior and country.

**Figure 2:**
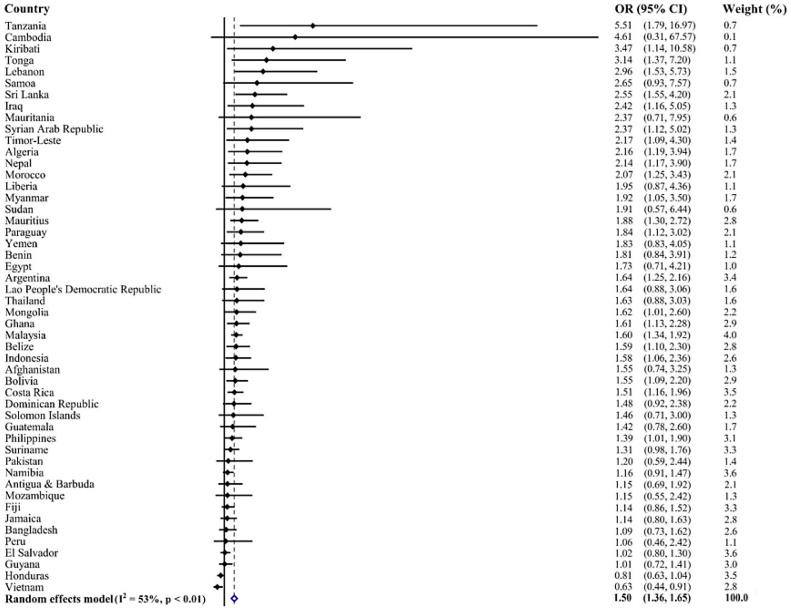
Association of physical education classes (≥3 days per week) with meeting the physical activity guidelines estimated by multivariable logistic regression. OR, odds ratio; CI, confidence interval. Models are adjusted for age, sex, food insecurity, and sedentary behavior. The pooled estimate was calculated by meta-analysis with random effects.

Fig. 2 shows the country-wise analysis of associations between physical education classes frequency (3 or more days) with meeting the PA guidelines. The analysis shows that adolescents with 3 or more days of physical education classes frequency were more likely to meet the PA guidelines. A moderate level of between-country heterogeneity was observed (*I*^2^ = 53.0%), with the overall estimate based on a meta-analysis being 1.50 (95% CI: 1.36–1.65).

## Discussion

This study represents an important addition to the literature. For the first time, there is a compiling of robust evidence on the association between physical education classes and PA, based on adolescents from 50 LMICs. Accordingly, this study has two major research findings; first, adolescents from LMICs with more physical education classes tend to be physically active (meeting the PA guidelines), regardless of sex. Secondly, although more physical education classes are positively associated with sufficient PA, this may vary across different countries with a moderate inconsistency.

Consistent with previous studies,^16^, ^17^, ^18^, ^19^ our study confirms that physical education classes are positively associated with adequate PA among adolescents. Indeed, a cross-sectional study with Estonian adolescents, using accelerometry to monitor physical education classes, found that adolescents had greater PA engagement than peers who did not take physical education classes. A similar study also measured PA level during physical education classes and daily PA amount,^17^ and highlighted that adolescents were more physically active when having more physical education classes, compared with their counterparts.^17^ Among Brazilian adolescents, a study also suggested that participating in more physical education classes was more likely with adequate PA.^24^ Indeed, the authors found that having physical education classes was associated with a higher level of PA in both sexes, in adolescents aged 11–19 years.^24^ Based on previous studies, our study affirms the positive role of physical education classes in improving the PA level among adolescents. The underlying mechanism linking physical education classes and PA may be related to the following plausible interpretations. First, any practice of PA, regardless of intensity, causes changes in the cerebral cortex and neurophysiological stimulation and can reduce the sensation of fatigue throughout the day, while improving mood and the willingness to keep moving.^13^ Another possible explanation is that physical education classes make young people familiar and more confident with physical activity, by gaining and developing the knowledge and skills for healthy lifestyles.^25^ Also, physical education class engagements can deepen the understanding of the importance of PA,^17^ which increases the possibility of engaging in additional activities out of school. However, discerning the actual mechanism linking physical education classes and PA among adolescents requires further empirical scrutiny.

The current study focuses on adolescents from LMICs, confirming the overall relationship of physical education classes and PA, and, indeed, represents a novel investigation. A noteworthy finding is that inconsistent with adolescents from high-income countries (HICs),^16^ our study suggests that the association between physical education classes and PA is not moderated by sex. Such a relationship has been supported by a study that included adolescents from LMICs,^16^ whilst among adolescents from HICs, the relationship between physical education classes and PA was most obvious in male participants.^16^ A possible explanation for these differences is the discordant strategies and policies to promote PA across HICs and LMICs.^10^, ^16^, ^24^ Indeed, prior research has observed that governmental actions, plans, and policies to encourage and promote PA are more evident in HICs, and some LMICs fail to have any government policies to increase PA, especially in the school environment.^9^

The present study found that there was a moderate inconsistency in the relationship between physical education classes and PA across LMICs. This finding suggests that, despite a significant positive relationship between physical education classes and PA, the relationship varies across countries. To our knowledge, this is the first study to investigate the between-country differences in the relationship between physical education classes and PA, among adolescents. Our results indicated that the role of physical education classes on PA promotion may be dependent on each country’s situation. Unsurprisingly, correlates/determinants of PA in different populations (multiple countries) may vary greatly;^26^, ^27^ therefore, the roles of physical education classes on PA may have relatively lower importance in explaining the variance. Although physical education classes have been encouraged as a vehicle to promote PA, and some countries have made physical education classes as a mandatory session in the school environment, some additional or unknown factors may also influence the effects of physical education classes on PA promotion (e.g. lacking facilities for physical education classes).^28^ Additionally, different countries have specific policies on physical education classes, which would exert an impact on the effectiveness of physical education classes. For example, the quality of physical education classes is lower to promise active time during the classes, and knowledge for active lifestyles would not be conveyed. These physical education classes may not be a way for PA promotion. However, this assumption goes beyond our research findings.

## Limitations, strengths, and future research

Although the present study represents a significant addition to the literature, the findings of this study should be interpreted with caution, in light of some inherent study limitations. First, owing to the nature of the cross-sectional study design, we cannot fully address the causality between physical education classes and PA among adolescents. Second, this study used self-reported measures to collect data on physical education classes and PA, which is subject to recall bias and may be a barrier to explore the relationship between physical education classes and PA among adolescents.^29^ Third, our study only includes a few control variables, which could influence our research findings. Indeed, such a limitation is attributable to sample size. If we included more control variables, some adolescents would be excluded, because they did not report the information on some control variables. Future research should address the study limitations for more robust evidence on the roles of physical education classes on PA promotion among adolescent populations in LMICs. Notwithstanding the limitations outlined above, the strengths of this study should be appreciated. We included the largest sample and data, from a multinational perspective, on physical education and PA in LMICs. Therefore, the research findings may have wider generalizability, which probably could be applied in global PA promotional efforts among adolescents. However, when generalizing the research finding, contextual differences and cautions should be mentioned carefully. Given the preliminary evidence from this study, the role of physical education classes impacting on PA must be further expanded. Future public health interventions or initiatives should consider physical education classes in health promotions for adolescents in LMICs.^30^

## Conclusion

This study offers multi-national evidence of the association between physical education classes and PA among a large sample of adolescents from LMICs. Our study also stresses the importance of physical education classes in increasing PA in adolescents. However, the association of physical education classes and physical activity among adolescents across different LMICs varies. Future longitudinal studies should confirm the veracity of the associations reported in this study. Nonetheless, with the incumbent evidence from the current study, when designing PA interventions for adolescents based on physical education classes, specific factors of each country should be taken into consideration.

## Funding

Si-Tong Chen is funded by the National Social Science Foundation of China (19CTY010).

## Conflicts of interest

The authors declare no conflicts of interest.
